# Microstructural white matter alterations in preclinical Alzheimer’s disease detected using free water elimination diffusion tensor imaging

**DOI:** 10.1371/journal.pone.0173982

**Published:** 2017-03-14

**Authors:** Andrew R. Hoy, Martina Ly, Cynthia M. Carlsson, Ozioma C. Okonkwo, Henrik Zetterberg, Kaj Blennow, Mark A. Sager, Sanjay Asthana, Sterling C. Johnson, Andrew L. Alexander, Barbara B. Bendlin

**Affiliations:** 1 Lieutenant, Medical Service Corp, United States Navy, Falls Church, Virginia, United States of America; 2 Department of Medical Physics, University of Wisconsin, School of Medicine and Public Health, Madison, Wisconsin, United States of America; 3 Waisman Laboratory for Brain Imaging and Behavior, University of Wisconsin, Madison, Wisconsin, United States of America; 4 Department of Radiology and Radiologic Sciences, Uniformed Services University of the Health Sciences, Bethesda, Maryland, United States of America; 5 Geriatric Research Education and Clinical Center, William S. Middleton Memorial Veteran's Hospital, Madison, Wisconsin, United States of America; 6 Wisconsin Alzheimer's Disease Research Center, University of Wisconsin, Madison, Wisconsin, United States of America; 7 Neuroscience Training Program, University of Wisconsin, Madison, Wisconsin, United States of America; 8 Wisconsin Alzheimer’s Institute, University of Wisconsin School of Medicine and Public Health, Madison, Wisconsin, United States of America; 9 Clinical Neurochemistry Laboratory, Department of Psychiatry and Neurochemistry, Institute of Neuroscience and Physiology, the Sahlgrenska Academy at University of Gothenburg, Gothenburg, Sweden; 10 UCL Institute of Neurology, Queen Square, London WC1N 3BG, United Kingdom; 11 Department of Psychiatry, University of Wisconsin, Madison, Wisconsin, United States of America; University of North Carolina at Chapel Hill, UNITED STATES

## Abstract

Brain changes associated with Alzheimer’s disease (AD) begin decades before disease diagnosis. While β-amyloid plaques and neurofibrillary tangles are defining features of AD, neuronal loss and synaptic pathology are closely related to the cognitive dysfunction. Brain imaging methods that are tuned to assess degeneration of myelinated nerve fibers in the brain (collectively called white matter) include diffusion tensor imaging (DTI) and related techniques, and are expected to shed light on disease-related loss of structural connectivity. Participants (N = 70, ages 47–76 years) from the Wisconsin Registry for Alzheimer’s Prevention study underwent DTI and hybrid diffusion imaging to determine a free-water elimination (FWE-DTI) model. The study assessed the extent to which preclinical AD pathology affects brain white matter. Preclinical AD pathology was determined using cerebrospinal fluid (CSF) biomarkers. The sample was enriched for AD risk (*APOE* ε4 and parental history of AD). AD pathology assessed by CSF analyses was significantly associated with altered microstructure on both DTI and FWE-DTI. Affected regions included frontal, parietal, and especially temporal white matter. The *f*-value derived from the FWE-DTI model appeared to be the most sensitive to the relationship between the CSF AD biomarkers and microstructural alterations in white matter. These findings suggest that white matter degeneration is an early pathological feature of AD that may have utility both for early disease detection and as outcome measures for clinical trials. More complex models of microstructural diffusion properties including FWE-DTI may provide increased sensitivity to early brain changes associated with AD over standard DTI.

## Introduction

Among the neuropathological characteristics associated with dementia due to Alzheimer’s disease (AD), neuronal loss and synaptic pathology—not β-amyloid plaques or neurofibrillary tangles—appear to be most strongly related to dementia severity and cognitive deficits in AD [1–5]. Beyond standard volumetric magnetic resonance imaging (MRI), there are few options for gauging cell loss, and sensitive approaches [5]are needed, especially to measure early changes. Diffusion-weighted MRI is an increasingly used technique that is sensitive to the random self-diffusion of water molecules. In an unstructured medium, diffusion characteristics reflect properties of the fluid such as temperature and viscosity. However, in structured media such as brain tissue, the measured signals are instead modulated by the geometry of the tissue microstructure [6]. Thus, water molecules are used as a noninvasive endogenous tracer to probe tissue microstructure.

Diffusion tensor imaging (DTI) describes the distribution of diffusion displacements using a Gaussian model and has been used extensively to assess tissue microstructure changes in aging, AD and preclinical AD [7–12]. Despite the promising sensitivity of DTI, it has known limitations that can impair utility and specific interpretation. To date, very few studies have applied advanced diffusion-weighted imaging methods for characterizing complex microstructural changes in the early stages of AD.

In this study, a free water elimination model was utilized to estimate and remove the signal contributions from cerebrospinal fluid and apparent free water components from the estimated diffusion tensor of the tissue. The FWE-DTI model contains an isotropic diffusion (free water) component with a diffusion coefficient that is constrained to be approximately three times larger than is typically encountered in tissue [13] in addition to the standard diffusion tensor. The free water diffusivity (3x10^-3^ mm^2^/s) matches the theoretical diffusion coefficient of CSF at body temperature [14]. The free water signal fraction, denoted as the *f*-value, corresponds to the water that has minimal interaction with tissue barriers over the diffusion time of the experiment. This model minimizes the CSF contamination of DTI measurements in tissues adjacent to CSF filled spaces such as the ventricles and cerebral cortex [15], which is particularly important as brain atrophy increases with aging and disease burden. Non-zero *f*-values present in tissues distal from CSF may also reflect the relative volume of extracellular spaces in the tissue [13,16,17].

We hypothesize that FWE-DTI would provide improved sensitivity to microstructural alterations that occur early in the development of AD. In addition to shedding light on the early features of AD pathogenesis, focusing on microstructural alterations is important given that plaques and tangles—while central features of AD—may be accompanied by additional features that predict progression to dementia [4,18,19]. Greater sensitivity in detecting microstructural pathology may help identify individuals at greatest risk for cognitive decline, as well as providing a novel outcome measure for clinical trials.

Thus, this study focused on individuals who may harbor preclinical pathology. Asymptomatic participants were recruited from the Wisconsin Registry for Alzheimer’s Prevention (WRAP) study[20], a cohort that is enriched for AD risk based on parental history of AD and a greater percentage of adults who carry a risk gene for AD, the ε4 allele of apolipoprotein E (*APOE* ε4)[21]. In addition to standard DTI participants underwent both hybrid diffusion imaging (HYDI) MRI [22] and lumbar puncture to assess levels of proteins related to AD and neurodegeneration in CSF. Biomarkers in CSF were used as a proxy for elevated preclinical AD pathology. CSF Aβ_42_ was used as a marker of cortical amyloid deposition; CSF Aβ_42_ predicts conversion to AD dementia [23] and correlates with in vivo [24] amyloid burden measured with positron emission tomography. Phosphorylated tau (P-tau_181_) was used as a marker of neurofibrillary tangle load [25], and total tau (T-tau) as a marker of degeneration of thin unmyelinated axons. Both P-Tau and T-Tau are elevated in patients with dementia due to AD [26,27], P-tau_181_ discriminates AD from non-AD dementias [28] and higher levels of T-Tau are associated with a more rapid disease progression [29]. Neurofilament light chain protein (NFL) measured in CSF was used as a marker of large caliber axon degeneration [30], while YKL-40 and monocyte chemoattractant protein 1 (MCP-1) were used as markers of microglial activation and neuroinflammation [31]. Finally, we evaluated soluble amyloid precursor protein beta (sAPPβ) as a marker of upstream APP processing in the β-secretase pathway [32]. We hypothesized that individuals with higher burden of preclinical AD pathology as shown by the CSF biomarkers would show microstructural alterations as measured by FWE-DTI.

## Methods

### Participants

Participants were 70 late-middle-aged adults (19 males and 51 females, age: 61.2 ± 6.2 yrs.) without dementia from the WRAP study. The WRAP cohort comprises well characterized and longitudinally followed participants who are either positive or negative for parental history of AD. Positive parental family history of AD classification was defined as having one or both parents with AD as determined by a validated interview [33] or autopsy-confirmed or clinically diagnosed probable AD as outlined by research criteria [34,35], and reviewed by a multidisciplinary diagnostic consensus panel. Detailed medical history obtained from phone interviews were conducted to confirm AD negative participants. Absence of family history of AD required that the participant’s father survive to at least age 70 years and the mother to age 75 years without diagnosis of dementia or cognitive deterioration. *APOE* ε4 genetic testing was performed at the Wisconsin Alzheimer’s Disease Research Center. Of those recruited 52 were identified as having a positive family history (FH+). Additionally, 27 subjects were carriers of one or more *APOE* ε4 alleles (APOE4+).

Participants underwent a comprehensive neuropsychological battery that included the Mini Mental State Exam [36]as a general cognitive screen, Rey Auditory Verbal Learning Test (RAVLT)[37], and the Weschler Memory Scale-Revised (WMS-R) [38]to assess memory function, in addition to Trail Making Test A and B [39], which reflect processing speed and executive function, respectively.

Study procedures were approved by the University of Wisconsin Health Sciences institutional review board and were in accordance with U.S. federal regulations. All participants provided written informed consent.

### Cerebrospinal fluid analyses

CSF was collected with a Sprotte 25-or 24-gauge spinal needle at the L3/4 or L4/5 using gentle extraction into polypropylene syringes. Samples were collected in the morning after a 12h fast. Approximately 22mL of CSF were combined, gently mixed and centrifuged at 2000g for 10 minutes. Supernatants were frozen in 0.5mL aliquots in polypropylene tubes and stored at -80°C. Samples were analyzed for T-tau, P-tau_181_ and the 42 amino acid form of amyloid β (Aβ_42_) using INNOTEST enzyme-linked immunosorbent assays (Fujiurebio, Ghent Belgium). Soluble α- and β-cleaved amyloid precursor protein (sAPPα and sAPPβ, respectively) and MCP-1 levels in CSF were measured using the Meso Scale Discovery technique (Meso Scale Discovery, Gaithersburg, MD, USA). YKL-40 was determined using a sandwich enzyme-linked immunosorbent assay (ELISA) (R&D Systems, Minneapolis, Minn., USA). CSF NFL was measured with a sandwich ELISA method (NF-light ELISA kit, UmanDiagnostics AB, Umeå, Sweden). Board-certified laboratory technicians who were blinded to clinical diagnosis performed all analyses on one occasion. All samples were analyzed according to protocols approved by the Swedish Board of Accreditation and Conformity Assessment (SWEDAC) using one batch of reagents (intra-assay coefficients of variation <10%). Individual biomarkers as well as biomarker ratios were entered into the statistical analyses as predictor variables.

### Magnetic resonance imaging

#### Acquisition

Diffusion-weighted imaging was completed on a 3-Tesla MR750 Discovery scanner (General Electric Healthcare, Waukesha, WI) using an 8-channel receive-only head coil. Separate diffusion scans were acquired for the DTI and FWE-DTI model fits. For the FWE-DTI scan, multiple nonzero b-values were acquired [40]: (number of images x b-value s/mm^2^): 7 x 0, 6 x 300, 21 x 1200, and 24 x 2700 s/mm^2^. For the DTI scan, 8 non-diffusion weighted volumes were acquired along with 40 diffusion encoding directions at a b-value of 1000 s/mm^2^ were acquired. Other pertinent parameters were: TR = 6500 ms (FWE-DTI) or 8000 ms (DTI), TE = 102 ms (FWE-DTI) or 67.8 ms (DTI), slice orientation: axial, slice thickness = 3 mm, and in-plane resolution = 2.5 mm x 2.5 mm interpolated to 0.9735 mm x 0.9735 mm.

#### MRI processing and analysis

Image analyses of both DTI and FWE-DTI used both region-of-interest (ROI) and voxel-wise methods. ROIs were generated using tractography methods. The primary outcome measures for DTI were fractional anisotropy (FA), a measure of directional water diffusion that is highly sensitive to microstructural features including axonal density, diameter, and myelination, and mean diffusivity (MD), a measure of isotropic diffusion that is sensitive to cellular structure, necrosis, and edema [6,41]. These were compared to FA and MD generated using FWE-DTI. Additionally, we conducted analyses on *f*-value generated with FWE-DTI, further described below. The processing steps for all data sets included movement and eddy current correction, gradient direction correction, and brain extraction before fitting the respective models. Eddy current correction and brain extraction was performed using the FSL toolkit.

#### FWE-DTI

The FWE-DTI signal model [42] is described by

$$S_{i}=S_{0}\left[\left(1-f\right)\exp\left(-b_{i}g_{i}^{T}Dg_{i}\right)+fexp\left(-bD_{iso}\right)\right]$$ (1)

where *S*_*i*_ and *S*_*0*_ are the signal from the i-th diffusion and non-diffusion weighted measurements, respectively, *D*_*iso*_ = 3 x10^-3^mm^2^/sec is the isotropic free water diffusivity, *D* is the tissue diffusion tensor, *b*_*i*_ and *g*_*i*_ are the diffusion-weighting amplitude (in mm^2^/s) and unit gradient encoding vector, respectively. In this implementation, the use of two non-zero b-values greatly simplifies the parameter space allowing accurate and stable diffusion measures even without spatial constraints and assumptions.

As separate scans were used for each of the diffusion models, all processing procedures through template construction were carried out in parallel, independent processing streams. The FWE-DTI and DTI models were fit in each subject’s native space. Subject specific templates were then created using DTI-TK http://www.nitrc.org/projects/dtitk/, as a means to leverage the full tensor information for optimal normalization[43–45].

#### Tractography

Whole-brain tractography was performed using the fiber assignment by continuous tracking (FACT) algorithm [46] as implemented in the Camino software package (http://cmic.cs.ucl.ac.uk/camino/). Tracts were seeded at the center of every voxel with an FA greater than or equal to 0.3. Specific tracts were then reconstructed by constraining viable fibers through the use of targeted inclusion and exclusion ROIs. An FA threshold of 0.3 and a curvature threshold of 60 degrees over 5 mm were used as stopping criteria. Visualization was carried out using TrackVis [47].

The tractography was carried out utilizing the tensor fitting from the FWE-DTI scheme. For each individual subject, the DTI scan was aligned to the native FWE-DTI scan. In this way, the DTI diffusion metrics could be projected onto the tracts reconstructed using FWE-DTI tensors. Using the FWE-DTI tensors instead of DTI tensors ensured a more full reconstruction of the fornix [48]. The metrics of interest for FWE-DTI were the FA, MD, and *f*-value. Standard DTI analyses were carried out for FA and MD. These metrics were calculated by taking the average value over all voxels, which intersected some portion of the reconstructed tracts.

**Fig 1:**
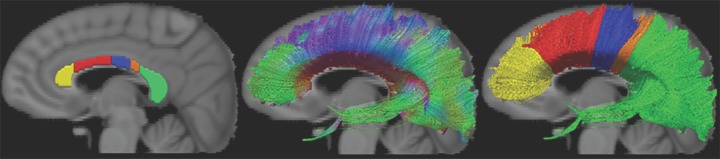
Seed ROIs and example segmentation of the corpus callosum overlaid on an MNI T1-weighted template. The CC was segmented into the following five regions: CC-I (yellow), CC-II (red), CC-III (blue), CC-IV (orange), and CC-V (green). The middle image shows a streamline reconstruction of the CC with DEC encoding based on the primary eigenvector direction. The rightmost image shows the same reconstruction with color determined by the seed ROI from which the tract originated.

**Fig 2:**
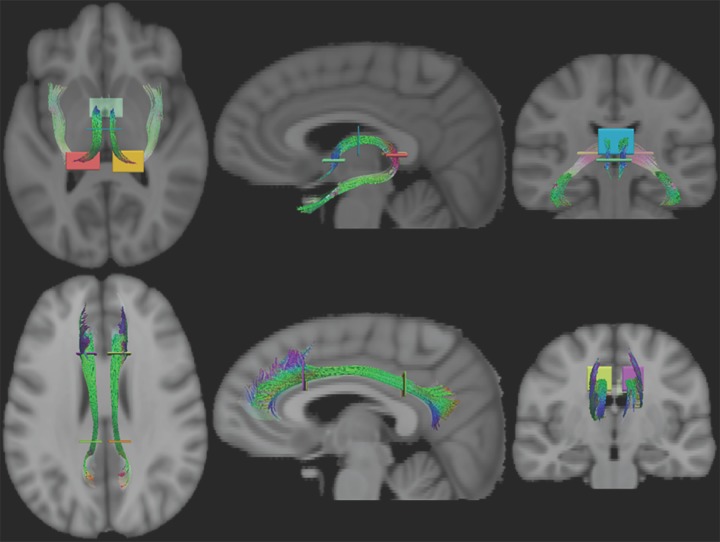
The ROIs used to define the fornix (top row) and superior cingulum (bottom row) in template space. Fornix tracts were reconstructed if they passed through the columns (green), body (blue) and either the right crux (red) or left crux (yellow). Cingulum tracts were reconstructed if they passed through an anterior ROI (left: yellow, right: purple) and a posterior ROI (left: orange, right: green).

ROIs were drawn in the template space and subsequently warped back to the native spaces to use as a seed point for deterministic tractography. Native-space tractography reconstructions were performed in brain regions known to be affected by AD [49–54], including the corpus callosum (CC), fornix, and cingulum. A single ROI defined on the midsagittal fractional anisotropy (FA) image was used to define the corpus callosum, Fig 1. This was further subdivided into five regions using the scheme proposed by Hofer and Frahm, based on fiber projection regions [55]. These regions were prefrontal (CC-I), premotor and supplementary motor (CC-II), primary motor (CC-III), primary sensory (CC-IV), and parietal, temporal, and visual (CC-V). The fornix was delineated based on intersection with two primary ROIs in the columns and body of the fornix and one of two secondary ROIs in the left and right crux [56], Fig 2. The superior portion of the right and left cingulum bundles were defined by tracts that pass through a pair of ROIs–anterior above the corpus callosum genu and posterior above the corpus callosum splenium [57,58], see Fig 2.

#### ROI statistical analysis

The mean value of each diffusion metric was extracted for each ROI for statistical analysis. For the fornix and cingulum bundles, the left and right structures that were separately defined above were merged created a single ROI for each structure. The metrics of interest were FA and MD for DTI as well as FA, MD, and *f*-value for the FWE-DTI acquisition. Analysis was carried out by fitting a generalized linear model in SPSS 22 (IBM Corp., Armonk, NY), treating the diffusion metrics as dependent variables and the CSF biomarkers and biomarker ratios as predictors, along with age and sex as covariates. In this way, the effect of each biomarker or ratio of markers was analyzed separately on each of the seven tracts and metrics of interest. As a means of controlling for multiple comparisons, a false discovery rate threshold of p ≤ 0 .05 was utilized [59].

#### Voxel-based analysis

In addition to the tractography analysis, a voxel-based analysis (VBA) was performed across white matter constrained by white matter mask. This mask was defined as all voxels for which FA ≥ 0.2. Given its importance in AD and susceptibility to early pathology, a separate VBA analysis was also conducted on the hippocampus using a mask based on the Desikan probabilistic structural atlas [60–63]. This atlas, which is in Montreal neurological institute (MNI) standard space, was warped to the population atlas space using an affine registration of an accompanying MNI space FA map to the population space FA map.

The VBA analysis utilized nonparametric permutation testing using the randomize function in FSL [64] along with threshold free cluster enhancement [65]. Prior to the VBA, all metric maps were smoothed with a 4mm full width half max Gaussian smoothing kernel. As with the tractography analysis, the effect of age and sex was controlled and the main effect of each biomarker on the diffusion metrics of interest was tested. For each permutation test, 25,000 permutations were carried out. Multiple comparisons were accounted for through the use of a family wise error threshold of p ≤ 0 .05. Once again, this analysis was carried out for the FWE-DTI FA, MD, *f*-value, and the DTI FA and MD. While all analysis took place in the population template space, these maps were warped to MNI via the inverse warp used for the hippocampus map for standardized reporting and visualization.

#### Cognitive analysis

A post-hoc analysis was performed to determine whether white matter impacted by preclinical AD pathology in turn underlies worse cognitive test performance. To test this, we used regression analysis to examine the relationship between white matter microstructure, and MMSE, Trails A, Trails B, RAVLT total, RAVLT delayed, and Wechsler Memory Scale total. As will be reported below, CSF biomarkers of AD were most closely associated with f-value, thus f-value served as the predictor variable in this analysis. To reduce the number of comparisons, we averaged the f-value extracted from all significant clusters and used multiple regression to test the relationship between mean f-value and the cognitive tests. Covariates were age, sex, education, and the time between MR scan and cognitive testing. Correction for multiple comparisons used Bonferroni correction, where there are 6 tests [.05/6], and a corrected p = .008.

## Results

### Demographics, cognitive function, and CSF

A summary of the cohort demographics is included in Table 1. Participants were non-demented. Mini-Mental Exam scores were largely in the range of normal, with the exception of one participant who scored 25 (3 scored 27, 8 scored 28, 16 scored 29, and the rest scored 30). The one participant who scored 25 on MMSE scored was within normal range on the Rey Auditory Verbal Testing delayed measure and thus remained in the analysis.

**Table 1:** Summary demographics, cognitive data, and CSF measures from the included participants.

Age	61.21 ± 6.16 yrs
Sex	18 male, 51 female
MMSE	29.34 ± 1.00
RAVLT Delayed	10.97 ± 2.85
RAVLT Total	52.74 ± 7.69
WMS-R Total	55.81 ± 14.69
Trails A	25.01428571 ± 8.02
Trails B	59.3 ± 22.15
sAPPα	640 ± 314 ng/mL
sAPPβ	531 ± 231 ng/mL
MCP	543 ± 119 ng/L
YKL	142076 ± 48926 ng/L
NFL	581 ± 191 ng/L
Aβ42	743 ± 194 ng/L
tTau	299 ± 112 ng/L
pTau_181_	41 ± 13 ng/L

Age was associated with NFL only, as shown in Table 2. One subject (male, APOE4+, FH-) was censured based on poor image quality along with an especially high value on the NFL assay that was more than 5.5 standard deviations above the group mean. This left 69 participants in the remainder of the analyses. Correlations with age in Table 2 were computed after censure of the subject noted above. Several CSF markers were correlated with one another with the strongest correlations being between sAPPα and sAPPβ (ρ = 0.954, P < 0.001) as well as pTau_181_and tTau (ρ = 0.876, P < 0.001). The APOE4+ group displayed a lower (P < 0.005) mean Aβ42 value (655 ± 168) compared to the APOE4- group (797 ± 192). None of the CSF markers were significantly associated with sex or parental family history of AD.

**Table 2:** Linear Pearson Correlation between biomarkers and age.

	sAPPα	sAPPβ	MCP	YKL	NFL	Aβ42	tTau	pTau_181_
Age	0.057	0.028	0.321	0.300	0.546†	-0.190	0.202	0.237
sAPPα		0.954†	-0.399*	0.385*	0.053	0.229	0.516†	0.631†
sAPPβ			-0.385*	0.436*	0.040	0.328	0.571†	0.663†
MCP				0.009	0.117	-0.241	-0.145	-0.318
YKL-40					0.193	0.299	0.517†	0.559†
NFL						0.095	0.319	0.314
Aβ42							0.188	0.230
tTau								0.876†

Significant correlations at P < 0.05 (*) and P < 0.001 (†) after Bonferroni correction are noted.

### Tractography

The diffusion metrics along each tract are presented in Table 3. All DTI results showed a positive correlation between the MD and CSF biomarkers (Table 4). Of the seven ROIs investigated, the ROIs situated in the posterior portions of the corpus callosum (CC-III, CC-IV, and CC-V) were the only ones which showed an association with CSF biomarkers, including pTau_181_, tTau, and YKL-40 as well as the ratio of each to Aβ42. The most robust results were for the ratios of markers as opposed to individual markers. Table 4 contains the results from tractography for relationships found significant at the uncorrected p = 0.05 threshold. This table also shows the corrected critical value significance threshold level once multiple comparisons are considered. No correlations were significant after false discovery rate correction.

**Table 3:** Diffusion metrics of tracts. All values are listed as mean ± standard deviation.

	FWE-DTI			DTI	
	F	MD (x10^-3^ mm^2^/s)	FA	MD (x10^-3^ mm^2^/s)	FA
CC-I	0.40 ± 0.024	0.042 ± 0.025	0.67 ± 0.038	1.09 ± 0.115	0.34 ± 0.073
CC-II	0.46 ± 0.048	0.40 ± 0.048	0.69 ± 0.045	1.18 ± 0.126	0.31 ± 0.059
CC-III	0.45 ± 0.037	0.38 ± 0.037	0.73 ± 0.049	1.14 ± 0.118	0.34 ± 0.058
CC-IV	0.47 ± 0.044	0.39 ± 0.016	0.71 ± 0.054	1.18 ± 0.142	0.33 ± 0.051
CC-V	0.45 ± 0.025	0.40 ± 0.019	0.76 ± 0.042	1.18 ± 0.108	0.38 ± 0.060
Fornix	0.72 ± 0.060	0.47 ± 0.022	0.72 ± 0.071	1.76 ± 0.206	0.25 ± 0.025
Cingulum	0.37 ± 0.029	0.42 ± 0.024	0.65 ± 0.079	1.02 ± 0.101	0.29 ± 0.055

**Table 4:** DTI tractography results.

Method	Tract	Metric	Predictor	Uncorrected P	FDR alpha
DTI	CC-IV	MD	pTau_181_ / Aβ42	0.0005	0.0002
DTI	CC-IV	MD	YKL-40/ Aβ42	0.0009	0.0005
DTI	CC-IV	MD	tTau / Aβ42	0.0010	0.0007
DTI	CC-III	MD	tTau / Aβ42	0.0011	0.0010
DTI	CC-III	MD	pTau_181_ / Aβ42	0.0017	0.0012
DTI	CC-IV	MD	pTau_181_	0.0022	0.0014
DTI	CC-III	MD	pTau_181_	0.0029	0.0017
DTI	CC-IV	MD	tTau	0.0036	0.0019
DTI	CC-III	MD	tTau	0.0038	0.0021
DTI	CC-III	MD	YKL-40 / Aβ42	0.0041	0.0024
DTI	CC-IV	MD	YKL-40	0.0058	0.0026
DTI	CC-V	MD	YKL-40 / Aβ42	0.0244	0.0029

All results with an uncorrected P ≤ 0.05 along with the false discovery rate α value.

For the FWE-DTI metrics, only the *f*-value showed a correlation with the CSF biomarkers (Table 5). As with DTI MD, the *f*-value was positively correlated with the CSF biomarkers. However, in this case the tracts that were affected were primarily the cingulum bundles and CC-I. The FWE-DTI *f*-value showed a positive relationship with pTau_181_, pTau_181_/Aβ42, tTau/Aβ42, YKL-40/Aβ42 and sAPPβ/Aβ42.

**Table 5:** FWE-DTI tractography results.

Method	Tract	Metric	Predictor	Uncorrected P	FDR
FWE-DTI	CC-I	f	pTau_181_ / Aβ42	0.0005	0.0002
FWE-DTI	Cingulum	f	pTau_181_ / Aβ42	0.0007	0.0003
FWE-DTI	CC-I	f	YKL-40 / Aβ42	0.0010	0.0005
FWE-DTI	CC-I	f	sAPPβ / Aβ42	0.0022	0.0006
FWE-DTI	Cingulum	f	sAPPβ / Aβ42	0.0024	0.0008
FWE-DTI	CC-I	f	tTau / Aβ42	0.0035	0.0010
FWE-DTI	Cingulum	f	pTau_181_	0.0051	0.0011
FWE-DTI	Cingulum	f	YKL-40 / Aβ42	0.0101	0.0013
FWE-DTI	CC-IV	f	pTau_181_	0.0114	0.0014
FWE-DTI	Cingulum	f	tTau / Aβ42	0.0202	0.0016
FWE-DTI	CC-III	f	pTau_181_ / Aβ42	0.0359	0.0017

All results with an uncorrected P ≤ 0.05 along with the false discovery rate α value.

### Voxel based analysis

The voxel based analysis revealed widespread areas where diffusion metrics were related to pTau_181_/Aβ42, tTau/ Aβ42 and sAPPβ/Aβ42 in both DTI and FWE-DTI analyses. For FWE-DTI but not DTI, pTau_181_ was significantly correlated with diffusion measures. As detailed in Tables 6 and 7, associations were largely found in temporal and frontal white matter. Similar to the tractography analysis, the only metrics which showed a significant relation to the CSF biomarkers were DTI MD and FWE-DTI *f*-value. Likewise, the relationship was positive for all of the clusters identified. An additional summary of the overall extent of significant findings may be found in Table 8.

**Table 6:** Listing of all significant clusters for which the FWE-DTI f-value was correlated to one of the predictors.

Biomarker	MNI Coordinats (x,y,z)	Peak T value	k (mm3)	Region
pTau_181_	(-41, -24, -8)	4.54	140	L inferior frontal-occipital fasciculus
pTau_181_	(-29, -23, 23)	4.67	136	L posterior corona radiate
pTau_181_	(-50, -31, -20)	4.81	128	L inferior temporal gyrus white matter
tTau / Aβ42	(-35, -48, -12)	6.69	3223	L fusiform gyrus white matter
tTau / Aβ42	(-20, 45, 5)	4.73	355	L anterior corona radiate
tTau / Aβ42	(-36, -76, -5)	5.15	69	L inferior occipital gyrus white matter
pTau_181_ / Aβ42	(-35, -48, -12)	7.94	31689	L fusiform gyrus white matter
pTau_181_ / Aβ42	(38, -40, -17)	5.64	1338	R fusiform gyrus white matter
pTau_181_ / Aβ42	(-36, -3, -31)	5.47	1178	L inferior temporal gyrus white matter
pTau_181_ / Aβ42	(36, -3, 24)	5.42	522	R inferior temporal gyrus white matter
pTau_181_ / Aβ42	(-35, -8, -7)	4.64	379	L insular gyrus
pTau_181_ / Aβ42	(17, 56, 10)	5.99	135	R superior frontal gyrus white matter
pTau_181_ / Aβ42	(40, 12, 21)	4.38	122	R inferior frontal gyrus white matter
pTau_181_ / Aβ42	(-8, -12, 40)	3.13	68	L cingulate gyrus
sAPPβ / Aβ42	(-36, -49, -12)	6.21	11941	L fusiform gyrus white matter
sAPPβ / Aβ42	(-15, 49, 10)	5.71	4845	L superior frontal gyrus white matter
sAPPβ / Aβ42	(38, -40, -17)	5.72	1572	R fusiform gyrus white matter
sAPPβ / Aβ42	(-27, 22, 28)	4.8	1562	L middle frontal gyrus white matter
sAPPβ / Aβ42	(-36, -3, -32)	6.05	1306	L inferior temporal gyrus white matter
sAPPβ / Aβ42	(-32, -11, 36)	5.16	1044	L precentral gyrus white matter
sAPPβ / Aβ42	(22, 52, 5)	5.11	547	R superior frontal gyrus white matter
sAPPβ / Aβ42	(37, 1, 27)	4.92	506	R precentral gyrus white matter
sAPPβ / Aβ42	(-17, -6, 48)	3.77	450	L superior frontal gyrus white matter
sAPPβ / Aβ42	(30, 19, 27)	4.26	395	R middle frontal gyrus white matter
sAPPβ / Aβ42	(27, 43, 5)	4.17	378	R middle frontal gyrus white matter
sAPPβ / Aβ42	(-13, -25, 54)	4.91	234	L precentral gyrus white matter
sAPPβ / Aβ42	(47, -38, 30)	4.55	137	R supramarginal gyrus white matter
sAPPβ / Aβ42	(-15, 0, 58)	3.7	81	L superior frontal gyrus white matter
sAPPβ / Aβ42	(-51, -8, 20)	4.82	78	L postcentral gyrus white matter

Information presented for each cluster includes diffusion metric and the related biomarker, relevant white matter region, MNI coordinates (in mm) of the peak t-value, and the cluster size (k). An explicit cluster size threshold is not needed when using TFCE as this threshold is strictly for reporting.

**Table 7:** Listing of all significant clusters for which the DTI MD was correlated to one of the predictors.

Biomarker	MNI Coordinats (x,y,z)	Peak T value	k (mm3)	Region
tTau / Aβ42	(-50, -21, -19)	5.39	3913	L parietal operculum
tTau / Aβ42	(24, 25, -13)	5.2	497	R posterior orbital gyrus white matter
tTau / Aβ42	(36, 2, -33)	5.26	381	R inferior temporal gyrus white matter
tTau / Aβ42	(54, -24, -16)	4.75	353	R middle temporal gyrus white matter
tTau / Aβ42	(37, -7, -5)	5.45	130	R insular gyrus
tTau / Aβ42	(35, 1, 19)	4.7	100	R precentral gyrus white matter
pTau_181_ / Aβ42	(-53, -33, -15)	6.36	5640	L inferior temporal gyrus white matter
pTau_181_ / Aβ42	(36, 1, -33)	5.8	491	R inferior temporal gyrus white matter
pTau_181_ / Aβ42	(28, 14, -8)	5.24	467	R inferior fronto-occipital fasciculus
pTau_181_ / Aβ42	(36, 3, 18)	5.5	351	R precentral gyrus white matter
pTau_181_ / Aβ42	(35, -6, 23)	5.13	255	R precentral gyrus white matter
pTau_181_ / Aβ42	(52, -21, -23)	5.04	194	R inferior temporal gyrus white matter
sAPPβ / Aβ42	(-53, -33, -15)	6.15	5342	L middle temporal gyrus white matter
sAPPβ / Aβ42	(51, -21, -23)	5.22	96	R inferior temporal gyrus white matter

Information presented for each cluster includes diffusion metric and the related biomarker, relevant white matter region, MNI coordinates (in mm) of the peak t-value, and the cluster size (k). An explicit cluster size threshold is not needed when using TFCE as this threshold is strictly for reporting.

**Table 8:** The total extent of significant voxels (mm^3^).

	pTau_181_	pTau_181_/Aβ42	tTau_181_/Aβ42	sAPPβ/Aβ42
FWE-DTI: f-value	404	35509	3701	25263
DTI: MD	~	7558	5398	5572

**Fig 3:**
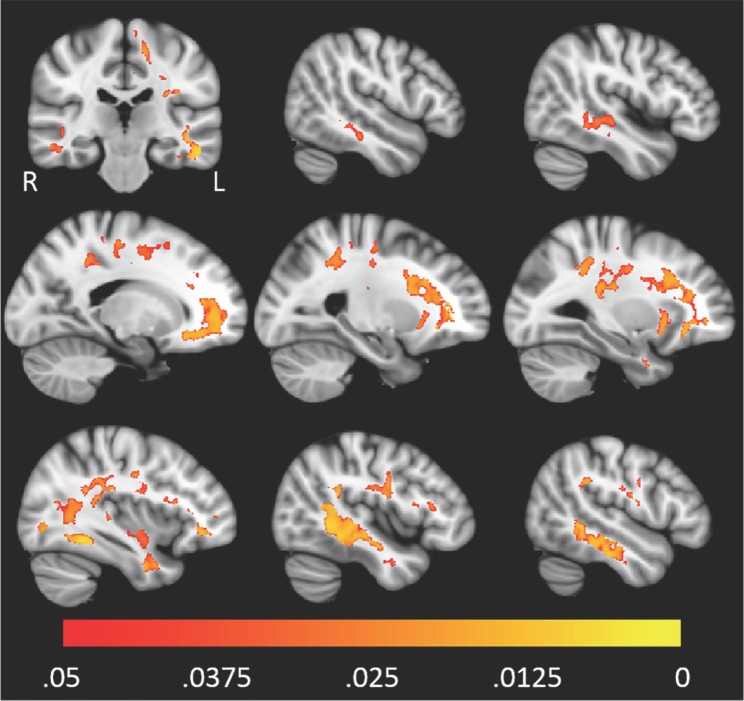
Higher levels of pTau_181_/Aβ42 were associated with higher FWE-DTI *f*-value throughout white matter. The red-yellow color scale above shows the familywise error corrected P-value. The underlay image is a T1w MNI template with 1 mm isotropic resolution.

**Fig 4:**
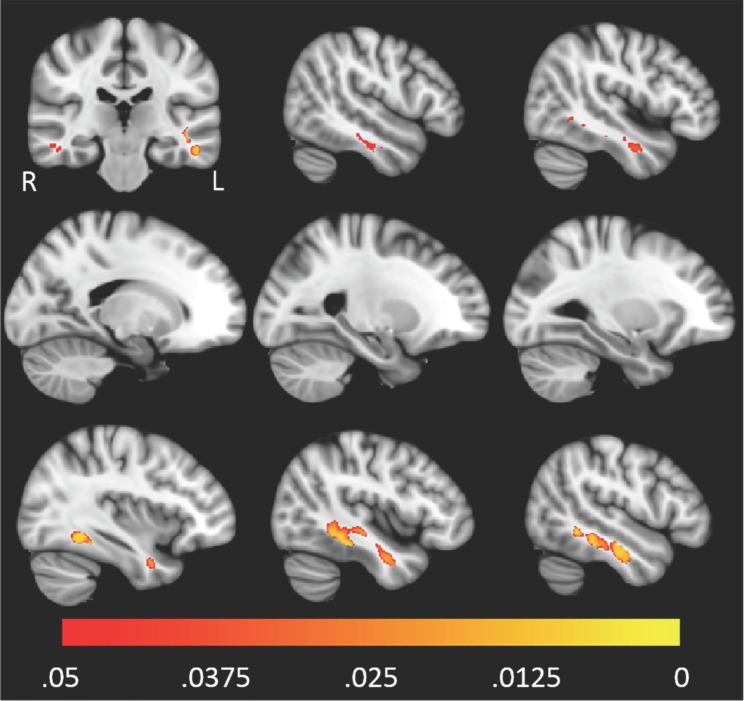
Higher levels of pTau_181_/Aβ42 were associated with higher DTI MD primarily in the left and right temporal lobes. The color scale, underlay, and presented slices are the same as those in Fig 3.

**Fig 5:**
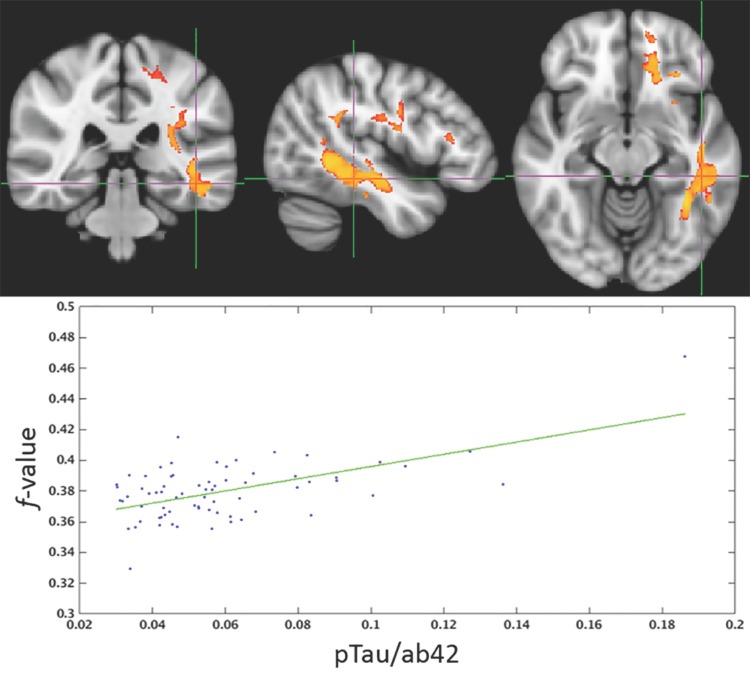
The largest contiguous cluster for which FWE-DTI *f*-value correlated with pTau_181_/Aβ42. All other clusters were masked out of the image. The color scale is the same as that of Fig 3.

**Fig 6:**
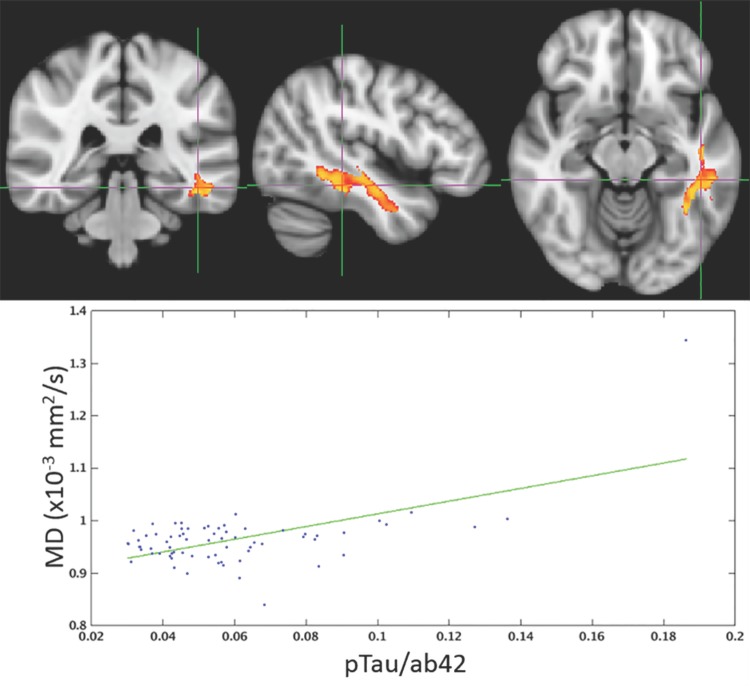
The largest contiguous cluster for which DTI MD correlated with pTau_181_/Aβ42. All other clusters were masked out of the image. The color scale is the same as that of Fig 3.

The spatial distribution of clusters for which FWE-DTI *f*-value or DTI MD were correlated with pTau_181_/Aβ42 is displayed in Figs 3 and 4, respectively. Scatter plots corresponding to the single largest clusters from Figs 3 and 4 are shown in Figs 5 and 6. For both imaging methods the largest cluster includes the left temporal lobe though with FWE-DTI the contiguous cluster is spread diffusely beyond. Regions where significant associations were found overlapped among comparisons. Tables 9 and 10 show the overlap between the significant cluster areas related to each biomarker or biomarker ratio. As shown in Table 9, with the exception of the clusters due to pTau_181_ alone, there was substantial overlap between all FWE-DTI clusters with one another; similarly, as shown in Table 10, significant regions of association in the DTI MD analysis also showed substantial overlap. No significant effects were observed for either diffusion method within the hippocampus.

**Table 9:** The percent overlap between the significant finding maps using the FWE-DTI *f*-value.

	f ~ pTau_181_/Aβ42	f ~ tTau/Aβ42	f ~ sAPPβ/Aβ42
f ~ pTau_181_	94%	36%	80%
f ~ pTau_181_/Aβ42		100%	80%
f ~ tTau/Aβ42			93%

These are computed as the percent of the smaller map, which overlaps with the larger map.

**Table 10:** The percent overlap between the significant finding maps using the DTI MD.

	MD ~ tTau/Aβ42	MD ~ sAPPβ/Aβ42
MD ~ pTau_181_/Aβ42	92%	85%
MD ~ tTau/Aβ42		66%

These are computed as the percent of the smaller map, which overlaps with the larger map.

### Cognitive function and white matter microstructure

Altered white matter microstructure was not a significant predictor of cognitive test performance. Of the six cognitive test scores assessed, only Trails B showed a modest relationship with mean f-value (b = -.27, p = .03), but this relationship did not survive correction for multiple comparisons.

## Discussion

An estimated 5.4 million Americans have AD. In the absence of effective treatments for the disease, there has been an increased focus on understanding the pathological features that occur in the earliest stages of the disease. Pathological features identified at an early stage may be targets for treatment prior to significant cell loss, in addition to providing utility for identifying candidates for clinical trials. In this study, we tested the extent to which preclinical AD involves alteration to white matter microstructure. White matter alterations have been observed in presymptomatic familial AD [66,67], in addition to being associated with risk factors for sporadic AD [51,68–70]. Thus, microstructural white matter changes may be an early and measurable feature of the disease, and new methods for modeling complex diffusion are expected to be sensitive to this early pathology. We found significant associations between markers of AD pathology measured in CSF, and white matter microstructure, especially as indicated by FWE-DTI *f*-value. In particular, elevated ratios of pTau/Aβ_42_ and tTau/Aβ_42_ were robustly associated with higher free water or *f*-value in frontal and temporal lobe white matter. A novel marker of the amyloid pathway (sAPPβ) also showed a robust association with altered microstructure as shown on FWE-DTI, especially when combined as a ratio with Aβ_42_. A similar correlation was seen with sAPPα as sAPPα and sAPPβ are themselves highly correlated.

It was previously shown in a smaller group of participants (n = 42) that tau and Aβ_42_ ratios are associated with MD[10]. Interestingly, the results of the current study also showed that MD derived from standard DTI was associated with higher pTau/Aβ_42_ in CSF, with peaks of association in temporal lobe white matter. Of note, the current study extends these findings by showing that FWE-DTI appears to demonstrate greater sensitivity to associations between AD pathology and white matter microstructure compared to standard DTI. Further, an analysis of hippocampal gray matter showed no association, suggesting that preclinical AD pathology may be detectable in white matter prior to changes in hippocampal microstructure. Gold et al [49] have previously tested the relationship between CSF markers of AD and both white matter alteration and hippocampal volume. While effects were observed in white matter, no relationship was observed between AD pathology as shown on CSF and hippocampal volume. The results of the current study suggest that indeed, effects on white matter may occur earlier than hippocampal alteration, and further, that this is the case even when diffusion weighted measures are used, which may be more sensitive to subtle change compared to T1-weighted imaging.

The FWE model has been applied previously to a variety of cohorts including older individuals with mild cognitive impairment [71–73]. In these instances, the model was applied with the goal of removing confounding CSF effects, as it was here. Some have referred to the f-value as an indicator of atrophy [74,75]. However, the results here indicate that the f-value may be something more than simply the CSF fraction. Parenchymal f-values reported here (~40%) are considerably higher than published reports from a similarly aged cohort (~7–9%) [72]. It is important to consider that the previously mentioned studies use a single b = 1000 s/mm^2^ diffusion acquisition and heavily regularized fitting scheme [76]. It is likely that the difference in f-value stems from the difference in acquisition schemes. Pasternak and colleagues have noted that using multishell acquisitions results in differences in estimated f-value [16]. While the difference presented here is much greater, so is the deviation in acquisition scheme. We also note that Bruggen et al. observed similar high free water values in their higher b-value multishell study of Alzheimer’s disease[77]. It is well documented that diffusion in tissue at high b-values is decidedly non-Gaussian [78], which may be a significant factor in the elevated f-value.

Non-zero *f*-values that are measured in tissue distal from CSF may reflect the relative volume of extracellular space in the tissue. In the context of AD, this signal could be due to loss of axons, or loss of myelin in brain white matter. Roher et al have demonstrated substantial alteration in both myelin and axons in familial and sporadic AD [79,80]. The pathology of AD includes hyperphosphorylation of tau protein, resulting in axonal abnormalities and displacement of tau to neuronal cell bodies [81]. Markers in this study that were related to tau (tTau and pTau), in addition to NFL, were strongly associated with white matter microstructural features as shown on FWE-DTI. Given that tau and NFL are components of the axonal cytoskeleton, these results may suggest that the *f*-value is sensitive to early axonal degeneration. CSF markers and advanced multi-compartment imaging are expected to shed further light on the temporal sequence of axonal and myelin alteration in the course of AD [82].

In this study, we found robust associations between CSF biomarkers reflecting core AD pathology, associated features, and white matter microstructure. Amyloid pathology has been linked with axonal degeneration. Hippocampal neurons cultured in vitro show axonal degeneration due to Aβ_42_ toxicity, an event that occurs prior to cell body death [83]. Other studies suggest that pathological changes in AD include leakage of amyloid from the extracellular space into the neuron, for example, at the axon hillock [84], causing subsequent axonal pathology. Krstic et al. review evidence that inflammation is involved in increasing neuronal cell vulnerability [85], and indeed, the results of the tractography analysis showed a positive association between MD and YKL-40, a marker of microglial activation—suggesting inflammation—as well as the ratio of YKL-40 to Aβ_42_. The results did not survive correction for multiple comparisons, but are suggestive. Additional studies are needed to determine the importance of biomarkers of inflammation for predicting development of dementia due to AD.

It is also of interest that higher sAPPβ/Aβ_42_ showed a widespread association with higher *f*-value across bilateral temporal and frontal white matter. Soluble Aβ-40 and Aβ-42 are elevated in white matter brain tissue assessed post mortem in AD patients compared to control [86]. APP cleavage by Beta-secretase 1 (BACE1) produces a membrane-bound fragment of APP that when further cleaved by γ-secretase yields several Aβ species including Aβ_42_ ultimately leading to formation of deposited amyloid plaques [87]. Products of the β-secretase pathway include increases in amyloid-β oligomers, which may be more toxic to neuronal cells than deposited plaques [88]. While CSF sAPPβ is not a direct measure of the oligomeric form of Aβ, the sAPPβ/Aβ_42_ ratio may reflect both increased proteolytic processing of APP through the amyloidogenic pathway, as well as increased deposition of amyloid, providing a better marker of amyloid pathology than either measure alone. The ratio may also account for inter-individual differences in APP production. To the author’s knowledge, this is the first study showing a link between elevated β-secretase cleavage of APP and *in vivo* white matter abnormalities as shown on diffusion-weighted imaging in humans.

Our study had a few limitations that deserve mention. The cohort was predominantly female (51 of 69 participants) with little male representation. Additionally, the parameters for the HYDI scan used for fitting the FWE-DTI model differs from the published “optimal” scan for this model [40]. As the DTI and FWE-DTI scans were independent, the acquisitions had different numbers of total images, as well as a different signal to noise ratio per image.

## Conclusion

The findings demonstrated that markers of AD pathology are associated with microstructural white matter alteration. In an improvement upon prior DTI studies, we employed a FWE-DTI approach to model complex water diffusion in cerebral white matter. Markers of elevated AD pathology were robustly associated with altered diffusion in bilateral temporal and frontal lobes, regions known to be involved in early stages of AD. These findings add to a growing body of literature indicating that white matter degeneration is likely an early and measurable feature of AD.
